# Histone modifications in the male germ line of *Drosophila*^a^

**DOI:** 10.1186/1471-213X-13-7

**Published:** 2013-02-22

**Authors:** Wolfgang Hennig, Alexandra Weyrich

**Affiliations:** 1 DAAD Laboratory, Shanghai Institutes for Biological Sciences, Chinese Academy of Sciences, Shanghai, China; 2 CAS-MPG Partner Institute for Computational Biology, Shanghai Institutes for Biological Sciences, Chinese Academy of Sciences, Shanghai, China; 3 present address: Institut für Genetik, Johannes Gutenberg-Universität Mainz, Mainz D-55128, Germany; 4 present address: Department of Evolutionary Genetics, Leibniz-Institute for Zoo and Wildlife Research (IWZ), Alfred-Kowalke-Str. 17, Berlin D-10315, Germany

## Abstract

**Background:**

In the male germ line of *Drosophila* chromatin remains decondensed and highly transcribed during meiotic prophase until it is rapidly compacted. A large proportion of the cell cycle-regulated histone H3.1 is replaced by H3.3, a histone variant encoded outside the histone repeat cluster and not subject to cell cycle controlled expression.

**Results:**

We investigated histone modification patterns in testes of *D. melanogaster* and *D. hydei*. In somatic cells of the testis envelope and in germ cells these modification patterns differ from those typically seen in eu- and heterochromatin of other somatic cells. During the meiotic prophase some modifications expected in active chromatin are not found or are found at low level. The absence of H4K16ac suggests that dosage compensation does not take place. Certain histone modifications correspond to either the cell cycle-regulated histone H3.1 or to the testis-specific variant H3.3. In spermatogonia we found H3K9 methylation in cytoplasmic histones, most likely corresponding to the H3.3 histone variant. Most histone modifications persist throughout the meiotic divisions. The majority of modifications persist until the early spermatid nuclei, and only a minority further persist until the final chromatin compaction stages before individualization of the spermatozoa.

**Conclusion:**

Histone modification patterns in the male germ line differ from expected patterns. They are consistent with an absence of dosage compensation of the *X chromosome* during the male meiotic prophase. The cell cycle-regulated histone variant H3.1 and H3.3, expressed throughout the cell cycle, also vary in their modification patterns. Postmeiotically, we observed a highly complex pattern of the histone modifications until late spermatid nuclear elongation stages. This may be in part due to postmeiotic transcription and in part to differential histone replacement during chromatin condensation.

## Background

During the first meiotic prophase, the chromatin of *Drosophila* male germ cells displays remarkable differences compared with most other organisms. In mitosis prophase chromatin typically condenses progressively into the microscopically visible individual chromosomes, which can be seen in their fully condensed state at metaphase. In most organisms this also holds true in meiotic prophase, when progressive chromosome compaction happens in different stages, accompanied by pairing and recombination. On the contrary in *Drosophila* males the characteristic meiotic prophase stages and recombination are missing [1]. Instead the chromatin goes through a stage of extreme decondensation until it rapidly condenses at prometaphase [2]. The meiotic prophase in *Drosophila* is also characterized by a high level of transcriptional activity which ceases shortly before the compaction of the chromatin and the entry into the first meiotic metaphase [3].

Condensation and decondensation of chromatin is to a large extent controlled or accompanied by both postranslational modifications of nucleosomal histones and sometimes by DNA methylation. While DNA methylation is insignificant in *Drosophila*[4-7], histone modifications are essential features of its chromatin. Modifications include methylation, acetylation and phosphorylation, preferentially in the N-terminal regions of histones H3 and H4, but also in histone H2A and H2B. The functional state of chromatin also depends on the substitution of histones by specific variants such as histone H3.3 [8-10] or the centromere-specific H3 variant CENP-A [11]. We previously identified two histone H3.3 variant genes in *Drosophila*, one of them strongly expressed in testes [10]. The histone H3.3 protein is highly enriched in the *Y chromosome* chromatin [12]. This histone variant is usually associated with highly transcriptionally active chromatin [13,14]. This observation, the special characteristics of the first meiotic prophase in *Drosophila* and the availability of antibodies specific for particular histone modifications [15] led us to study the patterns of histone modifications in the male germ line. Our observations reveal that the male germ line of *Drosophila* shows a characteristic pattern of histone modifications. We observe preferential methylation of H3K9 in the *Y chromosome* chromatin whereas H3K27 methylation is more prominent in the *X* and autosomal chromatin. We also observe that certain histone modifications are specific of spermatid nuclei. The observation of an absence of H4K16-acetylation in primary spermatocytes is consistent with the absence of dosage compensation during the transcriptionally highly active meiotic prophase as has been previously shown by the work of Rastelli and Kuroda [16] (see also the recent review: [17]).

The postmeiotic development of male germ cells of *D. melanogaster* was first described by Tates [18] and subsequently by Fuller [19]. The male germ line development of *D. hydei* was described by Grond [20] and Hennig and Kremer [21]. In postmeiotic cells chromatin displays complex patterns of condensation and decondensation [2]. At least part of the histones are replaced by protamine-like proteins [22-25], but some histones are maintained up to the final compaction stage in the mature sperm head [12]. The remaining histones in late spermatid chromatin have modifications. Acetylated histone H4 seems to be involved in histone to protamine transition [26]. The distribution of phosphorylated histone H4S1p and H4S10p has been described earlier by Krishnamoorthy et al. (Figure six in [27]. Our study reveals that the different histone modifications, as detected by highly specific antibodies, display a complex pattern during the development of the spermatid nucleus. Recently, a study of the enzymes involved in H3K9 methylation has been published by Ushijima et al. [28]. The conclusions presented seem in part to deviate from our observations but the cytology displayed by these authors is difficult to interpret and does not permit to relate to our observations.

## Results

Our study concerns modifications of histones H3 and H4 in germ and somatic cells of *Drosophila* testes. We used antibodies with a high specificity extensively documented in a series of publications from Dr. Jenuwein’s laboratory ([15,29-32] and others). Of the 19 antibodies directed against specific histone modifications two did not show significant signal in germ line or somatic cells of testes (anti-H3K4me1 and anti-H4K8ac, both of commercial origin, see Methods –data not shown). The other antibodies display substantial differences between different germ cell stages as well as the somatic cells of the testis envelope (summarized in Table 1).

**Table 1:** **Summary of the histone modification in testis of *D. melanogaster***

**Antiserum**	***Germline***			***Soma (testis)***	
	**Gonia**	**Spermatocytes**	**Spermatids**	**Polytene**	**Diploid**
H3K4me2				early	EU
H3K9me1	*	* increasing, NO	%		EU
H3K9me2	* HET °	* (+) decreasing	very early	-- ZERO --	HET °
H3K9me3	(*) #	Y chromosome °	%	HET + dots	HET
H3K9ac	(+)	Y chromosome °	early	EU	(+)
H3K27me1	(*)	X, autosomes		HET °	& EU
H3K27me2	(*)	X, autosomes		EU	EU
H3K27me3	HET	X, autosomes °	early %	EU	EU
H3K36me2	- ZERO -	(+)	-- ZERO --	EU	
H3K36me3				-- ZERO --	
H4K20me1	- ZERO -	X, autosomes °	early	EU (dots) °	EU (dots) °
H4K20me2	- ZERO -	°	early, asymmetric °%	EU	HET
H4K20me3	- ZERO -	increasing, NO	early%	-- ZERO --	EU
H4K5ac	##	(+)	(+)	-- ZERO --	
H4K12ac		X, autosomes °	°	(+)	
H4K16ac	early	-- ZERO --	early°	(+)	&
H3S10p		dots	°	early	

-- ZERO --: no reaction.

(…): parenteses: weak reaction.

#: hub cells strong reaction.

##: hub cells only.

*: cytoplasm.

Early: early developmental stages.

EU: euchromatin only.

HET: heterochromatin only.

NO: nucleolus strongly labeled.

PM: postmeiotic stages (according to [2].

&: particularly strong reaction in bulb cells.

%: nuclei during meiotic divisions labeled.

Dot: spotted label.

Increasing: increasing label intensity with age.

Decreasing: decreasing label intensity with age.

Asymmetric: decreasing from basis to tip of elongating spermatid nucleus.

°: shown in the figures.

### Male germ cells

All 17 modifications studied with exception of H4K16ac, were found in primary spermatocytes. We observed H3K9me2, H3K36me2 and H4K5ac only at low or very low levels (Table 1).

**Figure 1:**
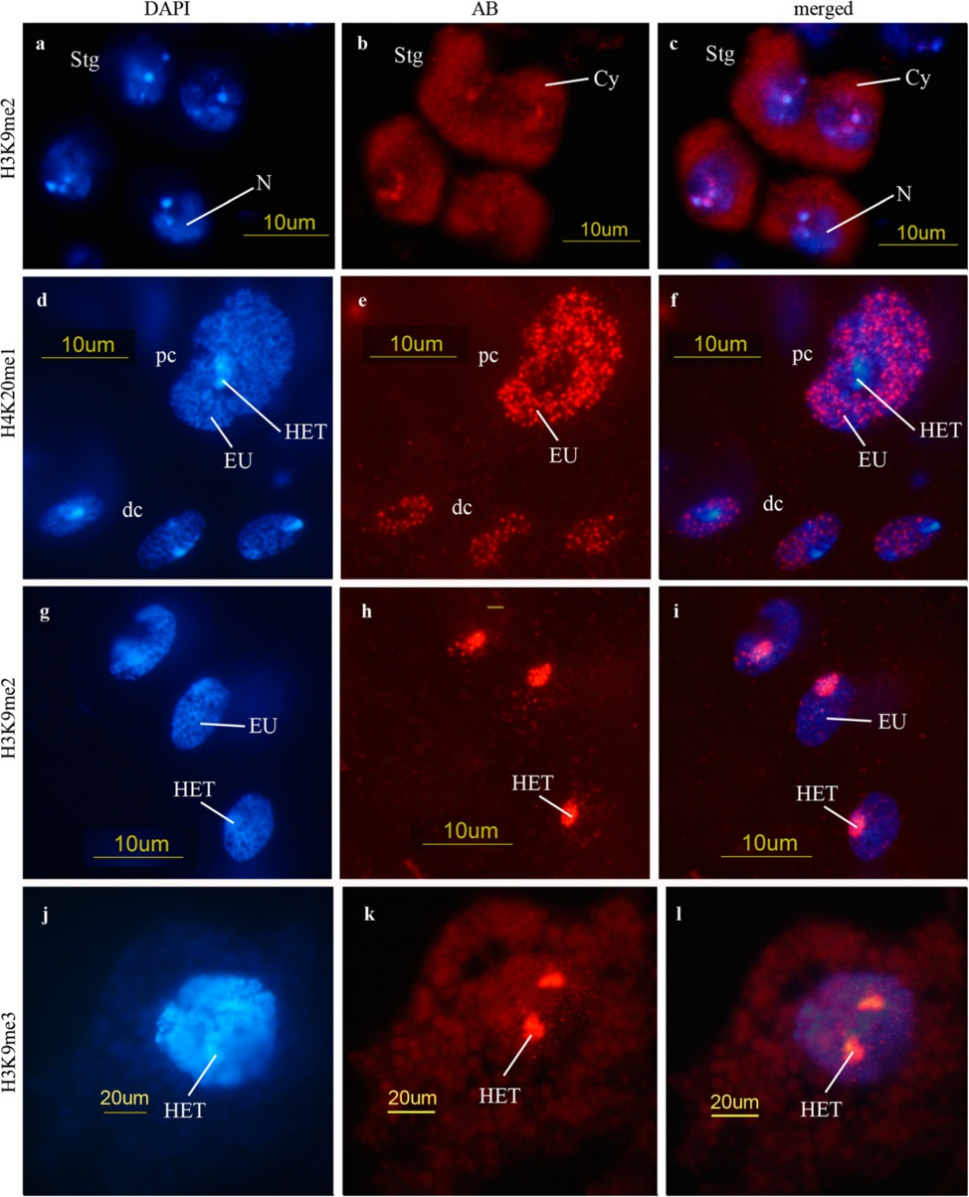
**Spermatogonia and somatic testis cells. **(**a**-**c**) H3K9me2; *D. melanogaster,* spermatogonia (Stg). Label in cytoplasm (Cy) and in heterochromatin (N: nucleus). (**d**-**f**) H4K20me1; *D. melanogaster,* small diploid cells of the testis envelope (dc) and one cell of low polyteny (pc). The label appears in a dotted pattern in the euchromatin (EU) while heterochromatin (HET) remains unlabeled. (**g**-**i**) H3K9me2; *D. hydei,* diploid cells of the testis envelope. Heterochromatin (chromocenter) and a few spots in the nucleus react with this antibody. (**j**-**l**) H3K9me3; *D. melanogaster,* polytene cell with labeled heterochromatin (HET). Also here, few spots appear in euchromatin. Cytoplasm and nucleus display background only. (**a**, **d**, **g**, **j**) DAPI; (**b**, **e**, **h**, **k**) antibody reaction; (**c**, **f**, **i**, **l**) merged pictures of DAPI and antibody.

The amount of H3K9me2 in germ cells is low. In spermatogonia this modification is mostly restricted to heterochromatin but it is also found in the cytoplasm (Figures 1a-c). The amount of H3K9me2 in spermatocytes decreases as meiotic prophase progresses. Postmeiotically H3K9me2 is restricted to very early spermatid nuclei (stages PM I-III -as defined in [2]). In diploid somatic cells of the testis envelope we find this modification restricted to heterochromatin (i.e. chromocenter, Figures 1g-i), whereas in polytene cells in the testis envelope it is totally absent.

The anti-H3K36me2 antiserum gives a very faint signal exclusively in primary spermatocytes. On the contrary in diploid somatic cells it is present at high levels all over the chromatin; in the testes polytene cells H3K36me2 is restricted to euchromatic chromosome regions.

H4K5ac is present at high level in the hub cells (compare with the H3K9me3 levels shown later (p. 9); these cells are located at the tip of the testis tube and comprise the germ line stem cells and the somatic cyst progenitor cells. In other germ cell stages H4K5ac was only detected at a much lower level. In diploid somatic cells H4K5ac is present all over the chromatin but polytene cells remain unlabeled.

**Figure 2:**
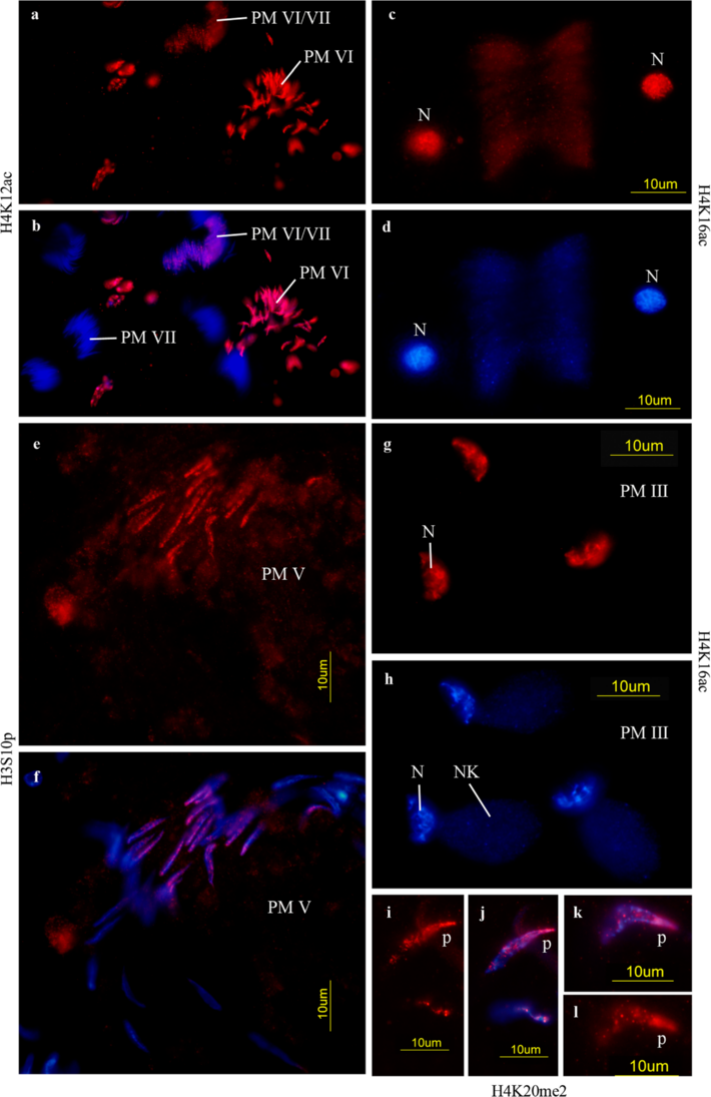
**Meiotic and postmeiotic cells. **(**a**, **b**) *D. melanogaster,* H4K12ac; late spermatid elongation stages (PM VI) and unlabeled sperm (stage PM VII). In the upper part elongating spermatids (PM VI/VII) with decreasing acetylated H4K12 are seen. (**c**, **d**) *D. hydei,* H4K16ac; two labeled daughter nuclei (N) after the first meiotic division. (**e**, **f**) *D. melanogaster,* H3S10p; elongating spermatids (PM V). (**g**, **h**) *D. hydei,* H4K16ac; young spermatids (PM III), very early elongation of the nucleus with chromatin in a restricted region. NK: Nebenkern, N: nucleus. (**i**, **l**) *D. hydei,* H4K20me2; early elongating spermatid nuclei (early PM V) with asymmetric label concentrated in the posterior part of the nucleus (p). (**a**, **c**, **e**, **g**, **i**, **l**) antibody reaction; (**b**, **f**, **j**, **k**) merged picture of DAPI and antibody; (**d**, **h**) DAPI. Staging of postmeiotic cells according to [2].

**Figure 3:**
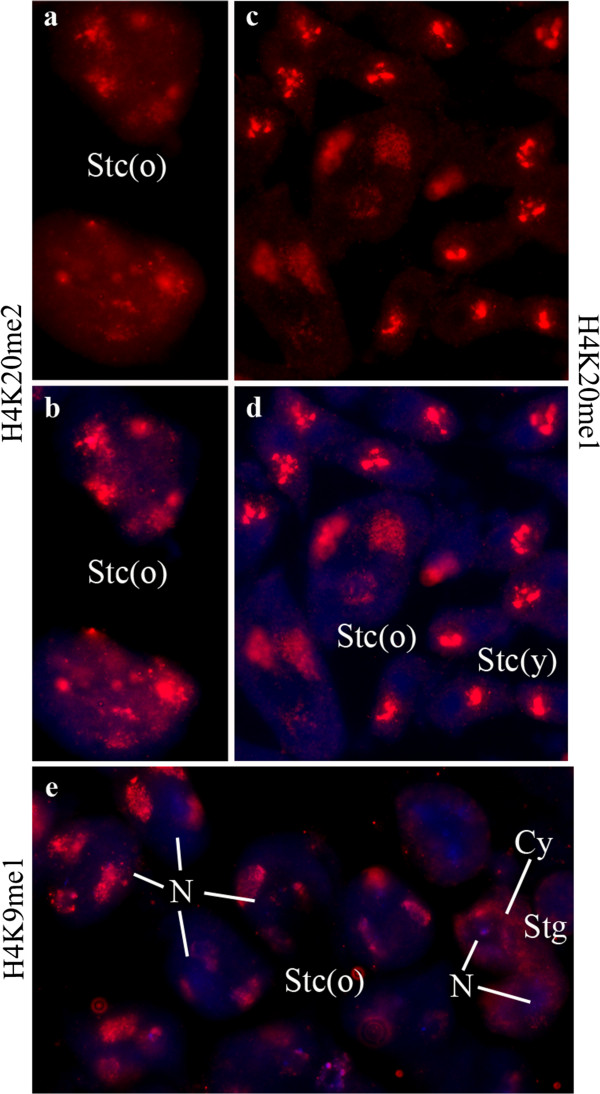
**Primary spermatocytes of *D. melanogaster. ***(**a**, **b**) H4K20me2; (**c**, **d**) H4K20me1; (**e**) H3K9me1. (**a**, **c**) Antibody reactions; (**b**, **d**, **e**) merged pictures of DAPI and antibody. (**c**-**e**) Show the characteristic patch-like patterns of prominent reactions with *X chromosome* and autosomes. In the smaller young spermatocytes (Stc(y)) the chromatin is more compact while in the larger nuclei of older stags (Stc(o)) is becomes more diffuse. In (**e**) two spermatogonia (Stg) are seen which still display some low cytoplasmic label (Cy) while the spermatocytes are not labeled around their nuclei (N). (**a**, **b**) display a more uniform label with some prominent patches in older spermatocytes. One of these patches represents the nucleolus.

H4K16ac behaves similarly as H4K5ac. It is present in spermatogonia (not preferentially in the hub cells) but it is not detected in primary spermatocytes^b^. H4K16 acetylation appears again during the meiotic divisions (Figures 2c-d) and is maintained in round spermatids (PM I-III according to [2]) (Figures 3g-h). Polytene cells show low levels of H4K16ac while diploid somatic cells show higher levels, in particular the somatic bulb cell nuclei of the testis tract.

All other histone modifications are found during the meiotic prophase and in diploid somatic cells, but they are missing in spermatogonia and polytene cells. Some of the modifications display very specific patterns (see Table 1).

A particularly remarkable observation is that H3 histones mono- and di-methylated in K9 are found in the cytoplasm of spermatogonia (Figures 1a-c). This cytoplasmic pool is present up to very early primary spermatocyte stages (Table 1). The amount of nuclear di-methylation in H3K9 decreases as the age of the spermatocyte increases and after meiosis only young spermatids (up to early nuclear elongation, states PM I-III according to [2]) display low levels of H3K9me2. The patterns of these histone modifications (H3K9me1/2) are similar to the ones observed for the distribution of the histone H3.3 variant [12].

**Figure 4:**
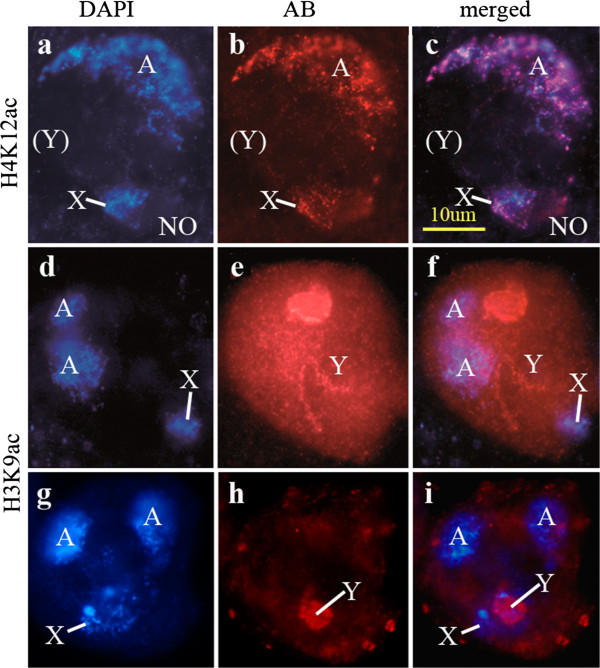
**Spermatocytes of *D. hydei *(a-f) and *D. melanogaster *(g-i). **(**a**-**c**) H4K12ac. Autosomes (A) and *X chromosome* (X) and nucleolus (NO) are stained. (**d**-**i**) H3K9ac. The *Y* chromosomal chromatin (Y) is stained. (**a**, **d**, **g**) DAPI; (**b**, **e**, **h**) antibody; (**c**, **f**, **i**) combination of DAPI and antibody.

We did not detect methylated H4K20 in spermatogonia but we detected them in later germ cells stages (primary spermatocytes: Figures 3a-d, spermatids: Figures 2i-l). In primary spermatocytes it is present in the chromatin of the *X chromosome* and the autosomes (Figures 3a, b). It is difficult to distinguish between the autosomes and the *Y chromosome* at this stage because its chromatin is strongly decondensed [2,33]. A *Y*-specific labeling can only be distinguished if a particular modification is either absent of present at very low level in the *X* and the autosomes (as for example, H3K9ac in primary spermatocytes -compare with the patterns shown in Figures 4d-i). H4K20me2 is restricted to elongating spermatids (stages as described in Figure 1o-p by Fuller [19] or PM V according to [2]). It is preferentially localized in the posterior region of the spermatid nucleus (Figures 2i-l), in contrast to the localization of other histone modifications which appear more uniformly distributed (cf. Figures 2a, b, e, f). H4K20me1 has an unusual pattern of distribution in spots in somatic cells (both diploid and polytene cells) while the heterochromatin remains unlabeled (Figures 1d-f). H4K20me3 was not detected in polytene cells, but is present in diploid envelope cells (Table 1). H4K20me2 H3K9me2 and H3K9me3 are restricted to heterochromatin in diploid cells (Figures 1g-i).

An unusual distribution was also observed for H3K9me3 and H3K9ac. Both modifications are found on the *Y*-*chromosome* lampbrush loops [34], but are absent from the *X* and the autosomes (Figures 4d-i). As mentioned before, identification of *D. melanogaster Y-chromosome* specific modifications is difficult due to the decondensed state of the *Y* chromatin [2]; this is particularly difficult when the other chromosomes are strongly labeled. We therefore used primary spermatocytes of *D. hydei* to confirm the presence of specific modification on the *Y chromosome* (H3K9ac in Figures 4d-f). The spermatocyte nuclei of *D. hydei* are larger and allow an easier distinction of the *Y* chromosomal lampbrush loops (for details see [35]) compared to *D. melanogaster*.

**Figure 5:**
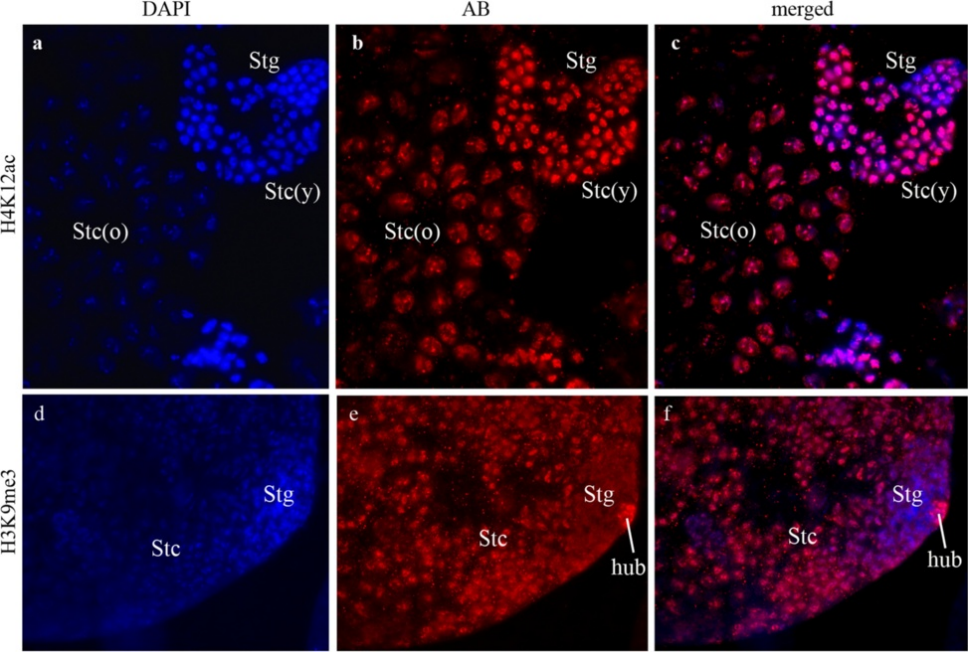
**Overviews of label in the testis tube of *D. melanogaster. ***(**a**-**c**) H4K12ac; different stages of primary spermatocytes show the gradual decondensation of the chromatin. In older spermatocytes (Stc(o)) mainly *X chromosome* and autosomes as well as the nucleolus are labeled. Younger spermatocytes (Stc(y)) and spermatogonia (Stg) have a more compact chromatin. The signal strength increases from spermatogonia to young spermatocyte stages (compare DAPI with antibody fluorescence!). (**d**-**f**) H3K9me3; apical tip of the testis tube. Spermatogonia are only slightly labeled and the cytoplasm seems slightly labeled (compare (e) with spermatogonia in (b)!). The hub cells (hub) display a strong reaction. Spermatocytes (Stc) show a more diffuse label compared with (b), as is characteristic for *Y* chromosomal chromatin label (cf. Figures 4d-i). Cytoplasm is unlabeled. (**a**, **d**) DAPI; (**b**, **e**) antibody; (**c**, **f**) merged pictures of DAPI and antibody.

The characteristic general picture of histone modifications in testis squashes is documented in Figures 5a-c. The figures displays the different patterns of H4K12ac in spermatogonia, early and late spermatocytes. In earlier developmental stages the labeling pattern is rather compact (corresponding to the more compact state of the chromatin); in contrast, in stages with strongly transcribed chromatin localized regions are visible with different compaction levels. Figure 5 also shows that the labeling patterns in different nuclei of cells of the same developmental stage are very uniform.

Figure 5d-f shows an example of hub cells strongly labeled for specific chromatin modifications (H3K9me3). In the case of H4K5ac the modification is specific of hub cells (see Table 1).

Some histone modifications are found in both round and elongating spermatids (Figures 2a-b) whereas others are restricted to the round spermatid stages (PM I and PM II according to [2]) (H3K9me2, H3K9ac, H3K27me3, H4K20me1, H4K20me2, H4K20me3, H4K16ac). H4K5ac is only present at a low level and H3K36me2 is not found in spermatids.

### Somatic diploid cells

Diploid somatic cells of the testis envelope display all histone modifications but with different patterns of distribution. Some modifications are restricted to heterochromatin (Figures 1g-i), some others to euchromatin (Figures 1d-f), and some occur in both eu- and heterochromatin (see Table 1).

Two histone modifications (H3K27me1, H4K16ac) are particularly abundant in the highly metabolically active bulb cells of the genital tract (Table 1).

### Polytene cells of the testis

Somewhat unexpectedly, polytene cells in the testis envelope appear to contain only certain specific histone modifications (see Tables 1 and 2). Some of these modifications are only observed at the early polytenization stages (H3K4me2, H3S10p). In polytene cells the variation in the distribution of modified histones in eu- and heterochromatin depends on the particular type of modification (Figures 1d-f, j-l) just like in diploid cells.

**Table 2:** **Comparison of histone modifications in *Drosophila *and mammals (modified from**[75]**)**

	***Drosophila *(Ebert 2006)**	**Mammals**	***Drosophila *testis**	***Drosophila *testis**
			**Polytene**	**Diploid**
H3K4me2	EU (IB)	EU	EU, HET	EU
H3K9me1	HET, EU (B)	EU	EU, HET	EU
H3K9me2	HET, EU (B)	EU	No reaction	HET
H3K9me3	HET, EU (B)	EU, fac. HET	HET	HET
H3S10p	EU (IB)	EU	EU	EU
H3K9ac	EU (B)	EU	EU	EU
H3K27me1	HET, EU (B)	HET, EU	HET	EU
H3K27me2	HET, EU (B)	EU	EU	EU
H3K27me3	HET, EU (B)	EU, fac. HET	EU	EU
H3K36me2	EU (IB)	?	EU	EU, HET
H3K36me3	EU (IB)	?	No reaction	EU, HET
H4K20me1	HET, EU (B)	EU, fac. HET	EU	EU
H4K20me2	HET, EU (B)	EU	EU	HET
H4K20me3	HET, EU (B)	HET	No reaction	EU
H4K5ac	?	?	No reaction	EU, HET
H4K12ac	?	?	EU, HET	EU, HET
H4K16ac	?	?	EU, HET	EU, HET

*B*: bands, *IB*: interbands, *EU*: euchromatin, *HET*: heterochromatin.

We also studied H3S10 histone phosphorylation. This modification is detected in all germ cell stages up to late spermatid elongation stages (at least PM V according to [2]) (Figures 2e-f); it is also present in diploid somatic and in early polytene cells (Table 1). Spermatocytes display a spotted distribution of this modification all over the chromatin.

## Discussion

Our investigation of histone modifications has shown a high degree of variation during the meiotic prophase. We have found some differences compared to the expected from the conventional “histone code” [36-39]. According to this some modifications are characteristic of inactive heterochromatin – essentially methylation of H3K9 and H3K27 [40,41] – and others of actively transcribed chromatin. The later is traditionally associated to several histone H3 modifications, in particular H3K4me, H3K36me, H3K9ac and H4K29me1. H4K20me1 is associated with transcribed chromatin while H4K20me3 is supposed to be involved in chromatin silencing [39]. It has recently emerged that histone patterns are more complex than previously expected and their specific relevance for gene regulation is far from being understood [42,43]. Earlier studies revealed that H3K9-methylation, a modification associated with inactive chromatin in pericentromeric heterochromatin, still permits transcription [44]. Most histone modifications are assigned to promoter regions [45], where histones with different modifications interact [46]. In ChIP experiments H3K27me3 and H3K4me3 were shown to co-localize in so-called bivalent domains, causing strong repression in embryonic stem cells [47]. In *Drosophila* the H4K16 acetylation can occur in interaction ("crosstalk") with H3K36me2/3 [48]. We have not observed the same in the *Drosophila* male germ line. Other species use different combinations in histone crosstalks [45]. The complexity of interactions between histones with different modifications has also been documented by Schübeler et al. [49]. These authors have shown that active chromatin is hyperacetylated in histone H3 and H4 and hypermethylated in K4 and K79 of histone H3; on the contrary, hypomethylation and deacetylation in those positions characterize silent chromatin. The level of the modification achieved is also relevant for the effects on chromatin activation or silencing.

A remarkable observation is the absence of H4K16 acetylation during the meiotic prophase of *Drosophila* males even though this stage is characterized by a high transcriptional activity [3,34]. H4K16 acetylation is important in the context of the dosage compensation mechanism in *Drosophila* males. MOF, MSL and MSL1-3 proteins form an RNP complex with roX1 and roX2 RNA regulating an increase in transcription of *X chromosomal* genes in males. MOF is a histone methylating enzyme and induces a high level of H4K16 acetylation [50-52] in the dosage compensated male *X* chromosome [53]. The absence of H4K16 acetylation during the first meiotic prophase suggests that during male meiosis no dosage compensation takes place. This has already been implied by earlier studies [16] and reviewed recently [17].

In this context the pattern of H3S10p histones becomes of interest. H3S10p has also been shown to be involved in dosage compensation by the formation of transcription-stimulating complexes with MOF [54]. It induces H4K16 acetylation in a crosstalk with H3K9ac [55] and additional factors [56], regulating dosage compensation. The absence of H4K16ac despite of the presence of the H3S10p histone in primary spermatocytes further supports the possibility that during the meiotic prophase no dosage compensation occurs. The restriction of H3K9ac to the *Y chromosome* in primary spermatocytes is an additional argument against dosage compensation in these cells. If dosage compensation takes place, it would be expected in the *X chromosome*[55]. In spermatogonia, where dosage compensation - as in all mitotic cells - is expected, H3S10p, H3K9ac and H4K16ac are expressed (Table 1). Strong arguments against dosage compensation in the male meiotic cells were also provided by Vibranovski et al. [57] from microarray data on the expression of *X* chromosomal genes.

An important role of H3S10p is its function in mitotic and meiotic chromosome condensation [58-60]. The presence in germ line cells and somatic cells of testes was therefore expected. Also in postmeiotic cells H3S10p should be required for chromatin condensation. This histone modification has been reported earlier in postmeiotic stages of spermatogenesis [27]. As H3S10p is also involved in early transcription initiation [61] it might play a role in postmeiotic transcription (which has more recently been reported [57,62]). The presence of most of the modified histones (in particular of H3K4me1/2, H3K9ac, H3K36me3, H4K12ac and H4K16ac) as general transcriptional marks in elongating spermatid nuclei (at least in stages PM I-V according to [2]) supports such a view. In this context it is also relevant that histone H3.3, a histone variant associated with highly transcribed chromatin [10,13,14], was found in elongating spermatid nuclei [12]. H3K9-dimethylation is not found in the H3.3 histone variant as opposed to other modifications characteristic of active chromatin [14]. This is consistent with our observation that H3K9me2 remains at a very low level throughout spermatogenesis and is restricted to very early, round spermatid nuclei (Table 1). In somatic cells of testis H3K9me2 is restricted to heterochromatin (Figure 1g-i).

Our observations on modified histones in spermatid nuclei agree to a great extent with earlier reports [25,26]. The differences observed may be due to different specificities of the antisera. The antisera provided by Jenuwein’s laboratory have a higher specificity for the particular modification than most if not all commercial antisera [15]. We also studied some modifications not included in earlier studies. Different modifications do not show identical patterns. Some of the modified histones are only specifically found in round and early elongating spermatid nuclei (H3K9me2, H3K9ac, H3K27me3, H4K20me1/2/3, H4K12ac, H4K16ac) (stages PM I-III according to [2]). In addition, H4K20me2, often associated with heterochromatin, displays a defined localization pattern within the spermatid nucleus (Figures 2i-l). This might be related to the patch-like gradual condensation of the chromatin in these nuclei [2,22]. Some of the histone modifications restricted to the earlier elongation stages usually reside characteristically in transcribed chromatin.

As it happens in other organisms, during *Drosophila* spermatogenesis, histones are replaced by more basic proteins [22-26]. The uneven distribution of H4K20me2 displays a remarkable correlation to the initiation of chromatin condensation as reported by Hauschteck-Jungen and colleagues [22]. However the disappearance of (modified) histones in our immunofluorescence experiments, does not prove the absence of histones in the mature sperm head. In our experience, it is very difficult - if not impossible - to achieve a detection of epitopes by immunocytology in the tightly condensed chromatin of mature spermatozoa. In some cases removal of the DNA from the nucleosomes is required to make the targeted epitopes accessible for binding [63]. To which extent histones are postmeiotically replaced by other proteins remains an open question. At least some histones persist to late elongation stages (Figures 2a-b). Transcription has been documented in these stages [57,62] and this may require the presence of modified histones. Whether these are subsequently removed from the mature sperm chromatin remains unclear.

The observed histone H3 modifications can be related to our earlier studies showing evidence of strong expression of one of the two histone H3.3 variants in the male germ line [10,12]. Immunocytology demonstrated that the variant histone H3.3 is not present in the nuclei of spermatogonia but is prominent in primary spermatocytes and postmeiotic chromatin (Figures six to ten in [12]). Histone H3.3 is a major chromatin component of these stages. In primary spermatocytes, histone H3.3 was found in *Y chromosome* chromatin. Histone H3.3-specific signal was observed in the cytoplasm of spermatogonia (Figure six(a) in [12]). This agrees with the patterns of the H3K9 histone modifications observed in our recent study. The distribution patterns of H3K9ac, H3K9me1, H3K9me2 and H3K9me3 can be correlated with the distribution of the histone H3.3 variant (Table 1). Methylated H3K9 histones are found in the cytoplasm of spermatogonia (H3K9me1/2 (Figures 1a-c) and in the *Y chromosome* chromatin (H3K9me3) (cf. Figures 4d-i). It implies that the variant histone H3.3 is modified in the cytoplasm and substitutes the cell-cycle regulated histone H3.1. The distribution patterns of H3K9me1/2/3 may reflect a successively increasing methylation level (Table 1). Although H3K9 methylation is generally assumed to be found in silent chromatin, exceptions have been reported [64] (see also below). The *Y chromosome* is highly transcriptionally active [3,65,66]. Histone H3.3 is found in transcriptionally active chromatin where it is incorporated in a replication-independent process [13,14]. In earlier methylation studies no special attention has been given to methylation of H3.3 and its effects. In combination with H3K9ac it might associated with the H3.3 variant in transcribed chromatin.

The decreasing level of H3K9me2 displays a remarkable correlation to the decreasing intensity of transcription in spermatocyte stages III and IV which was documented by autoradiography [3]. As H3K9me3 would promote the inactivation of the transcription this could suggest that H3K9me2 could be converted into H3K9me3 at the onset of chromatin compaction in the primary spermatocyte nucleus towards the end of the meiotic prophase.

In our earlier studies with H3.1- and H3.3-specific antisera, histone H3.1 signals could preferentially be assigned to autosomes and the *X chromosome* Figures six(e, f) in [12]. This location coincides with the immunofluorescence signals obtained with methylated H3K27 and H4K20 antibodies. Spermatocytes display high levels of H3K27me3 and H4K20me1, and lower levels of H3K27me3 and H3K27me1. H3K9me1 is present at lower levels on all chromosomes. The signal increases with the age of the spermatocyte. The same age-dependent increase was also found for H3K27me1. This might be related to an increasing conversion of K27methylation into H3K27me2 and later into H3K27me3.

The lower level of H3K9-methylation (in particular H3K9me2) is not unexpected as H3K9-methylation is generally assumed to be associated with transcriptionally inactive chromatin, while H3K4 methylation is preferentially associated with transcriptionally active genomic regions [67]. However, H3K9 is not exclusively found in inactive chromatin as revealed by studies of Vakoc et al. [64,68] and Loyola et al. [43]. Their conclusions agree with our observations. The highly active state of the meiotic prophase chromatin in *Drosophila* as documented by autoradiography and hybridization data [3,65,66] also contradicts the conventional idea that not only methylation of H4K20, but also of H3K27 correlate directly with large-range chromosomal repression of gene activity. On the other hand, the typical mark for active chromatin, H4K16ac, was not detected in meiotic prophase cells. This acetylated histone was however observed in nuclei during the meiotic divisions and is much stronger in early spermatid nuclei (Figures 2c-d, g-h). The presence of H3K4me2, H3K9ac, and H3K36me3 in spermatid nuclei, usually considered as indication of transcribed chromatin, suggests that it is present in decondensed chromatin. We have earlier observed that chromatin in spermatid nuclei passes through a condensation-decondensation cycle [2]. H3S10p is involved in the activation of transcription in combination with H4K16ac and H3K9ac. The transcription in elongating spermatid nuclei might be controlled by this mechanism [7,57,62].

We observed relatively strong cytoplasmatic signals for methylated H3K9 (Figures 1a-c) in spermatogonia. In primary spermatocyte nuclei, methylated H3K9 is found associated with the *Y chromosome* (Table 1) as is histone H3.3. This might indicate an initial methylation in gonial cytoplasma and subsequent transfer into the spermatocyte nucleus. According to Loyola et al. [43] mono- and di-methylated H3K9 is the only histone modification that has been found in a non-nucleosomal state. The tri-methylated state is achieved in a subsequent step. Moreover, methylation of K9 in H3.a and H3.3 follow different pathways. Since patterns of distribution closely agree, we assume that the cytoplasmic representation of H3K9me1/2 resembles that of the histone H3.3 variant.

Whether the very weak cytoplasmatic signals of H3K27me1/2 are real or reflect background signals remain an open question. The reaction in polytene cells (Figure 1j-l) argues in favor of background. For both modifications only one charge of antiserum was available, therefore we could not determine whether the background was an artifact of the antiserum (for other antisera, different charges were studied, see Methods). According to [43] H3K27 methylation takes exclusively place in nucleosomal constitutions.

An increasing level of the modification in primary spermatocytes was observed for H3K9me1 and H4K20me3 (Table 1). Remarkably, both modifications were not only found in the general chromatin but also associated with the nucleolus (Table 1). In somatic cells both modifications are associated with euchromatin. This is consistent with the high transcriptional activity in the primary spermatocyte nucleolus. No other histone modifications were seen in nucleolar chromatin of the male germ line. In our earlier studies no reactions with total histone were seen in nucleoli [12]. The amount of nucleosomes in nucleoli is small as has been shown by electron microscopy [69,70]. The small amount of histone seems to be highly marked with H3K9me1 and H4K20me3. The increasing amount of H4K20me3 may be related to the decreasing transcription of rDNA in old spermatocyte nuclei (stage III of *D. hydei*: [3]) as shown by audioradiography.

Histone H4 associated with H3.1-containing nucleosomes (Table 1) was mainly acetylated at K12 and K16. H4K12ac reacts mainly with autosomes and the *X chromosome* in primary spermatocytes. This suggests that - in agreement with our conclusions drawn from the location of methylated H3K27 and H4K20me1 histones - histone H3.1 is deposited preferentially in the *X chromosome* and autosomes while histone H3.3 resides mostly in the transcribed *Y* chromosomal lampbrush loops.

It has been postulated that the *X chromosome* becomes inactivated during the male meiotic prophase [71,72]. As in earlier studies [2] the DNA patterns in primary spermatocyte nuclei show no evidence of the presence of heterochromatin and our present observations support this conclusion that inactivation of the *X chromosome* in meiotic prophase is unlikely. This conclusion is supported by molecular data on the transcription of *X* chromosomal genes [10,57]. Arguments against an *X* inactivation have also been discussed by McKee and Handel [73] and Meiklejohn et al. [74]. The arguments in favour of *X* inactivation during the meiotic prophase of *Drosophila* are based on rather indirect genetic evidence. However cytological and molecular data do not support such a mechanism.

Also the pattern of modified histones in somatic cells of the testis deserves some attention due to deviation from patterns typically observed in other cell types (Table 2). The absence of histones H3K9me2, H3K36me3, H4K20me3 and H4K16ac in polytene cells is unexpected in view of the reports of Ebert et al. [75] (Table 2). However, localized histone modifications spread over single bands or interbands in giant chromosomes as they are typically observed in salivary gland chromosomes [75] might be difficult to detect at low degrees of polyteny as present in testis cells. We have not found published data confirming that salivary gland giant chromosomes at low levels of polyteny display modification patterns similar to those of high polyteny. The modification patterns reported for polytene chromosomes might only be achieved at higher degrees of polyteny.

## Conclusions

The investigation of the histone modifications in *Drosophila* testes revealed an unexpected diversity of patterns. While almost all types of methylation and acetylation studied are present, they display stage-specific or even chromatin-specific locations. The expression of H3K9-methylated histones coincides with the location of the histone variant H3.3. In particular H3K9me3 and H3K9ac are preferentially associated with the transcribed regions of the *Y chromosome* in primary spermatocytes. H3K27- and H4K20-methylated histones, on the other hand, are primarily associated with active autosomal and *X chromosome* chromatin as is H4K12ac. Most remarkably, H4K16ac is not found in primary spermatocytes while H3S10p is observed at a low level. H3K9ac is essentially restricted to *Y chromosome* chromatin (Figures 4d-i) but not significantly detected in the *X chromosome*. Since these chromatin components participate in the *X* chromosomal dosage condensation, our observations suggest that during the first meiotic prophase dosage compensation in the *X chromosome* does not take place. Our data also indicate different modification patterns of histones in postmeiotic stages. While some modifications are restricted to early round spermatid nuclei, others persist almost up to individualization, when a final condensation of the chromatin occurs. In particular H3K9me1 and H3K27me1 are detected in later elongating spermatid nuclei. Both histone modifications are also found in euchromatin of somatic diploid cells of the testis. Their presence in late elongating spermatid heads is probably related to postmeiotic transcription documented in late spermatid heads. We had earlier shown that the histone H3.3 variant at this stage disappears from the spermatid nuclei. Although no histone reactions were seen in mature sperm nuclei, it cannot be decided by immunological methods whether histones persist in mature sperm chromatin as the compaction might render epitopes inaccessible.

## Methods

### *Drosophila* strains

We used a *Drosophila melanogaster CS* and the *white*^*1118*^ strain from the collection of the Institute of Genetics, Johannes Gutenberg-University, Mainz/Germany. *Drosophila hydei* and the wild type strain belong to our own collection.

### Preparation of slides and immunocytology

Testes were dissected in testis buffer (0.047M NaCl; 0.183M KCl; 10mM Tris pH6.8) [3] squashed and fixed in testis buffer with 3.7% para-formaldehyde or 4% formaldehyde (Merck) and subsequently frozen in liquid nitrogen. After removal of the cover slip, slides were immersed in methanol at −20°C for at least 5min, transferred into 1/1 (v/v) methanol/acetone at −20°C for 5min and finally into acetone at −20°C for 5min. They were washed twice in PBS with 0.1% Triton-X100 at room temperature and fixed in PBS with 3,7% para-formaldehyde or 4% formaldehyde for 20min at room temperature. After washing twice for 10min in PBS the slides were blocked for at least 30min in PBS with 1% bovine serum albumin (BSA, Sigma) and incubated with 7μl of the respective primary antibody diluted in PBS, at 4°C in a humid chamber overnight. The next day slides were washed 3x in PBS including 1% Triton-X100. Incubation with the secondary antibody diluted in PBS took place for 1h at room temperature in a humid chamber. Finally slides were washed 3x in PBS with 0.1% Triton-X100 at room temperature and embedded with antifading solution (1% 1,4-phenylenediamine in 50% glycerol in PBS). Inspection of the slides was done with a Zeiss Imager Z1 with a Plan-Neofluar 40x/1.3 Oil, a Plan Apochromat 63x/1.4 Oil or a Plan-Apochromat 100x/1.4 Oil, or with a Nikon Eclipse E600 epifluorescence microscope with Plan Fluor 40x/0.75 and Plan Apo 100x/1.40 Oil DIC H optics.

The primary antibodies [15] against the different methylated histones H3 and H4 were affinity-purified rabbit antibodies kindly provided by Dr. T. Jenuwein, IMP Vienna, (diluted 1:1000 in PBS): H3K9me1 #4858, H3K9me2 #4679, #4677 and 2^nd^ bleed, H3K9me3 #4861 and 2^nd^ bleed, H3K27me1 #8835, H3K27me2 #8841, H3K27me3 #6523 and 2^nd^ bleed, H4K20me1 #0077, H3K20me2 #0080, H4K20me3 #0083. Additional antibodies against modified histones were H3K4me1, from Abcom (Cat.No. ab 8895), H3K4me2 from Abcom (Cat.No. ab 7766), H3K36me2 from UBI (Cat.No. 07–274) and H3K9ac from UBI (Cat.No. 06–942) and H4K8ac of unknown origin (provided by Dr. D. Schweizer).

As secondary antibodies, the Alexa 488 – labeled ″Goat-Anti-Mouse″ - F(ab’)2 fragment of IgG (H+L) (Molecular Probes; Cat.No. #A11017) or the Dylight-547 or the Dylight-549 – conjugated Goat-Anti-Rabbit IgG (H+L) (Pierce; Cat.No. #31020) – were applied in a dilution 1:200 in PBS.

## End notes

^a^A preliminary version of this paper was published in [1]. A Weyrich: DNA methylation, histone modification and the transcription factor dE2F1 in Drosophila. Mainz: Johannes Gutenberg-University Mainz; 2007.

^b^ Some authors refer to young primary spermatocytes as “premeiotic”. This may lead to wrong interpretations as all primary spermatocytes must correctly be considered as meiotic cells. G1 and S-phases in spermatocytes are very short and most spermatocytes reside therefore in meiotic prophase I.

## Authors’ contributions

Both authors designed the project. The microscopy was done by WH, slides were prepared by both authors and the manuscript was written by both authors. Both authors have approved the final manuscript for publication.
